# Physicochemical, Stress Degradation Evaluation and Pharmacokinetic Study of AZGH101; a New Synthesized COX2 Inhibitor after I.V. and Oral Administration in Male and Female Rats

**Published:** 2018

**Authors:** Hoda Bahmanof, Simin Dadashzadeh, Afshin Zarghi, Alireza Shafaati, Seyed Mohsen Foroutan

**Affiliations:** a *Department of Pharmaceutics, School of Pharmacy & Protein Technology Research Center, Shahid Beheshti University of Medical Sciences, Tehran, Iran. *; b *Department of Medicinal Chemistry, School of Pharmacy, Shahid Beheshti University of Medical Sciences, Tehran, Iran.*

**Keywords:** Pharmacokinetic, Ketoprofen, Selective COX-2 inhibitors, AZGH101, Wistar rat

## Abstract

Nonsteroidal anti-inflammatory drugs (NSAIDs) act mainly via inhibition of prostaglandins synthesis by inhibition of cyclooxygenase (COX) isoenzymes (COX-1 and COX-2). Selective COX-2 inhibitors which are also known as coxibs provide the main therapeutic effects of NSAIDs. Zarghi *et al*. reported 6-benzoyl-2-(4-(methylsulfonyl) phenyl) quinoline-4-carboxylic acid (AZGH101) as a novel derivative of ketoprofen with improved selectivity index (COX-1/COX-2 inhibitory potency) in comparison with ketoprofen. In this study, the log P and stability of AZGH101 were evaluated and the pharmacokinetic characteristics of this compound were investigated following intravenous (10 mg/kg), and oral administration (20 mg/kg), to Wistar rats. As the data demonstrated, the AZGH101was classified as lipid soluble compound and had suitable stability according to forced degradation protocol ICH guideline for new drug substance. This derivative absorbs, distributes, and eliminates similar in both sexes. The AUC 0-∞, absolute bioavailability, Cl, and Vd were not different in both sexes. According to the obtained data, the AZGH101 does not have a sex dependent pharmacokinetic in Wistar rats.

## Introduction

Traditional none steroidal anti-inflammatory drugs (NSAIDs) such as naproxen, ibuprofen, ketoprofen, and diclofenac are one the most prominent class of drugs that was used in the world (1, 2). However, these drugs do not contain steroids in structure; they exert similar effects to steroids (3). These drugs have been prescribed and sold as over the counter drugs for over a hundred years (3, 4). It was reported that over 30 billion doses were sold as OTC in the USA in 2001 (4). NSAIDs have been prepared to mimic the pharmacological effects of aspirin and provided anti-inflammatory, antipyretic, and analgesic effects (5). This class of drugs acts mainly via inhibition of prostaglandins synthesis by inhibition of cyclooxygenase (COX) isoenzymes, inhibition of tumor necrosis factor (TNF)-α, and inducible nitric oxide synthase (iNOS) (3, 4). 

It is reported that there are three COX isoenzymes; COX-1, COX-2, and COX-3 (2, 6, 7). Although all NSAIDs inhibited COX isoenzymes, the degree of inhibition varies. COX-1 is responsible for maintenance of physiological functions of the human body, especially gastrointestinal mucosa (2, 3, 7). COX-2 is an inducible isoenzyme which is located in macrophages and involved in inflammatory processes (2, 3, 7). COX-3 is similar to COX-1 and exerts its antipyretic effect when inhibited by acetaminophen (3, 6). 

NSAIDs can be divided into traditional nonselective NSAIDs such as ibuprofen, indomethacine, and ketoprofen which inhibit both COX-1 and COX-2 isoenzymes and selective COX-2 inhibitors which are also known as coxibs (2, 3, 5, 7). The main therapeutic effects of NSAIDs are related to COX-2 inhibition while the gastrointestinal and renal disorders are related to COX-1 inhibition (2-5, 7). Moreover, it is reported that inhibition of COX-2 reduces the risk of colorectal, breast and lung cancers, prevention of osteoporosis and slow the progress of Alzheimer’s disease (4, 8). Coxibs such as celecoxib, rofecoxib, and valdecoxib are introduced as selective COX-2 inhibitors to the market but some of them such as rofecoxib and valdecoxib are withdrawn from the market due to their adverse cardiovascular side effects (2, 7). These reasons provide a rationale for design and synthesis of new selective COX-2 inhibitors. Zarghi *et al*. reported that quinoline ring is a very suitable scaffold for COX-2 inhibitory activity and introduced the new derivatives of ketoprofen as novel class of selective COX-2 inhibitors in (2010) (2, 9). They reported 6-benzoyl-2-(4- (methylsulfonyl) phenyl)quinoline-4-carboxylic acid (AZGH101) compound with IC50 of 0.077 (µM) for COX-2 (9). The tendency of this new derivative of ketoprofen against inhibition of COX-2 is about 10 times greater than ketoprofen (10). Moreover, the selectivity index (COX-1/COX-2 inhibitory potency) of AZGH101 is improved in comparison with ketoprofen (9, 10). 

In this study, the physicochemical properties of AZGH101 were entirely evaluated and the pharmacokinetic characteristics of this compound following intravenous (IV) and oral administration to male and female rats were investigated. 

## Experimental

*Materials*

**Figure 1:**
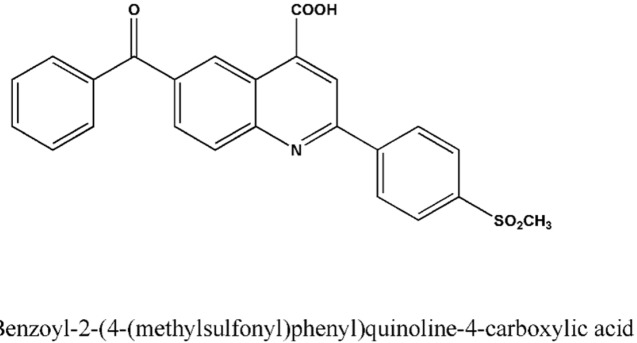
Chemical Structure of AZGH101.

The compound AZGH101 (6-benzoyl-2-(4-(methylsulfonyl) phenyl)quinoline-4-carboxylic acid) (Figure 1) as novel derivative of ketoprofen was synthesized and purified in the institute of authors (9). Acetonitrile and methanol as analytical grade were purchased from Merck (Darmstadt, Germany). Ultrapure water was obtained from Millipore Direct-Q system. All other chemicals and solvents were of analytical grade and provided from Merck (Darmstadt, Germany). Internal standards (IS) were obtained as a gift from the analytical laboratory of author’s institute.

*Structure and purity*

A UV spectrum of AZGH101 was acquired by Shimadzu 1200 spectrometer (Japan) at a wavelength of 200-400 nm in the methanol. An Infra Red (IR) spectrum with KBr disk was acquired using a Termoelectron Co. Model Nicolet 380 spectrometer (USA). An 6410 Agilent LCMS triple quadrupole mass spectrometer with an electrospray ionization (ESI) interface was used for mass spectral measurement. Melting point was determined with Electro Thermal 9200 capillary apparatus (Japan). Elemental analysis was performed for C and H.

*HPLC Analysis*

*Apparatus and chromatographic conditions*

The Knauer chromatographic system consisted of a Wellchrom 1001 pump, a Wellchrom K-2700 Diode Array Detector, a Wellchrom solvent degasser and a Rheodyne injector with 20 μL loop. Instrument control, data acquisition, and analysis were performed by Chromgate software version 3.1. The separation of analyte was done on the MZ C18 (250 × 4 mm, 5 µm) column from Merck (Darmstadt, Germany) at ambient temperature (25 ºC). The detector wavelength was fixed at 266 nm.

*Preparation of standard solutions and samples*

The working standard of AZGH101 and ISs were prepared in methanol at 1 mg/mL concentration and stored at 2–8 ºC. 

Quality control (QC) samples were prepared at 20, 40, 80, 160, and 320 ng/mL concentrations by dilution of working standard in mobile phase.

Analytical samples were prepared by dilution of working standard in either mobile phase or in spiking with blank plasma. 

*Analytical method development*

Method development was involved investigation of mobile phase constitutions, flow rates (1.5-2 mL/min) changes and mobile phase pH (2-4) on peak resolution and separation. As a mobile phase, either acetonitrile or methanol in varying ratio (v/v) was added to 10 mM buffer phosphate.

*Plasma sample preparation*

Plasma samples were prepared by precipitating method. Different precipitant agents were used and the efficacy of them were compared. In brief, precipitants (acetonitrile, perchloric acid 12 and 24% and/or combination of NaOH 1 N and zinc sulphate 0.7 M) were added to plasma samples in different ratios and were shaked for 2 min. The obtained suspensions were centrifuged for 10 min at 10000 rpm and the supernatants were separated.

*Analytical method validation*

This method was validated according to ICH analytical method validation guideline for specificity, intra and inters -day precision, accuracy, limits of detection (LOD) and quantification (LOQ), linearity and stability.

The specificity was evaluated by comparison between blank samples and spiked ones with either AZGH101 or IS. 

For linearity study, the calibration curves were constructed for AZGH101 according to peak areas of five concentrations between 20 and 320 ng/mL with 5 replicates, by linear regression, without weighting.

For intra-day precision the relative standard deviation (RSD) of 3 replicates for quality control (QC) samples were calculated. The RSD of these samples were calculated in 3 replicates over 3 days to establish inter-day precision.

The difference between true value and measured concentration of 5 replicates for QC samples was used for accuracy determination.

The LOD was defined as the lowest concentration of analytes which produced response 3 times higher than background noise. The LOQ was also defined as the lowest concentration of analytes which could be determined with the RSD of 20% and accuracy within ± 20.

In the recovery study the spiked biological samples were prepared by the precipitating method. The obtained samples were then analyzed by chromatographic method and compared with the similar spiked samples in methanol. Samples were diluted in mobile phase where necessary.

*Physicochemical properties*

Melting point was determined with an Electrothermal 9200 apparatus (UK). Aqueous solubility and octanol/water partition coefficient of the synthesized derivative was determined according to Organisation for Economic Co-operation and Development (OECD) Guideline for Testing of Chemicals, No.105, and No. 107 respectively (11-13).

**Table 1:** HPLC validation parameters for AZGH101 in methanol and plasma

	**Plasma**	**Methanol**
Calibration range (ng/mL)	160-2560	20-320
Calibration points	5	5
Correlation coefficient (r)	0.9987	0.9999
Slope	2957	0.007
Intercept	62.44	0.615
Limit of quantification (LOQ) (ng/mL)	70	12
Limit of detection (LOD) (ng/mL)	30	4
Precision (RSD)^a^ Intra (n = 3)		
Level 1	0.11	0.36
Level 2	0.55	1.28
Level 3	2.54	0.69
Inter (n=9)		
Level 1	0.11	0.36
Level 2	0.29	1.28
Level 3	2.58	0.69
Accuracy (%)^a^		
Level 1 Level 2 Level 3	100.04 99.95 100.34	100.04 100.27 99.95

a : In plasma level 1 = 2560 ng/mL, level 2 = 640 ng/mL, level 3 = 160 ng/mL and in methanol level 1 = 320 ng/mL, level 2 = 80 ng/mL, level 3 = 20 ng/mL.

**Table 2:** Forced tests results for AZGH101. The number of replication was 3.

**Stress Condition**	**Mean ±SD** ^a^	**RSD** ^b^
Hydrolysis(Acid)	0.95±0.02	2.10
Hydrolysis(Base)	0.92±0.03	3.26
Hydrolysis (Buffer pH=7)	0.94±0.02	2.13
Oxidative stress (H2O2-3%)	0.93±0.04	4.30
Oxidative stress (H2O2-30%)	0.91±0.02	2.19
Thermal Degradation (solid phase)	0.96±0.03	3.12
Photodegradation (Acid)	0.94±0.02	2.13
Photodegradation (Base)	0.94±0.01	1.06
Photodegradation (Buffer pH=7)	0.95±0.02	2.11

a : Standard deviation

b : Relative standard deviation

**Table 3:** Pharmacokinetic parameters (mean ± SD) of AZGH101 following oral (20 mg/kg) and intravenous (10 mg/kg) administration to Wistar rats

	**Oral route**			**Intravenous route**		
	**Female**	**Male**	***P *** **value**	**Female**	**Male**	***P *** **value**
β (h-1)	0.13 ± 0.01	0.14 ± 0.01	NS	0.355 ± 0.006	0.349 ± 0.008	NS
Ka (h-1)	1.00 ± 0.10	0.92 ± 0.18	NS	-	-	-
α (h-1)	3.86 ± 2.99	3.34 ± 1.26	NS	3.70 ± 0.80	4.25 ± 0.99	NS
t1/2 Ka (h)	0.70 ± 0.07	0.78 ± 0.16	NS	-	-	-
t1/2 β (h)	5.34 ± 0.48	4.99 ± 0.52	NS	1.97 ± 0.06	2.01 ± 0.04	NS
t1/2 α (h)	0.24 ± 0.10	0.23 ± 0.07	NS	0.20 ± 0.04	0.17 ± 0.05	NS
Tmax (h)	0.50 ± 0.00	0.50 ± 0.00	NS	-	-	-
Cmax (μg/mL)	21.83 ± 1.33	20.39 ± 1.49	NS	-	-	-
AUC 0-∞ (h.μg/mL)	41.33 ± 4.20	42.77 ± 2.24	NS	79.41 ± 5.81	77.12 ± 3.62	NS
Vd (mL)	245.87 ± 44.86	233.55 ± 27.82	NS	89.26 ± 7.68	94.08 ± 6.42	NS
Cl (mL/h)	31.73 ± 3.31	32.45 ± 1.65	NS	31.63 ± 2.25	32.48 ± 1.57	NS
F (%)	27.73 ± 0.01	26.02 ± 0.03	NS	-	-	-
C0 (μg/mL)	-	-	-	86.77 ± 11.32	92.33 ± 11.02	NS

**Figure. 2:**
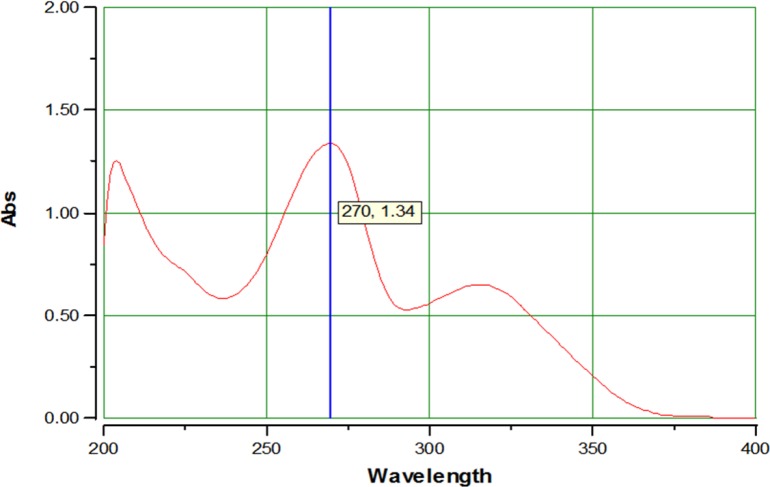
UV spectrum of AZGH101 from 200-400 nm in methanol.

**Figure 3:**
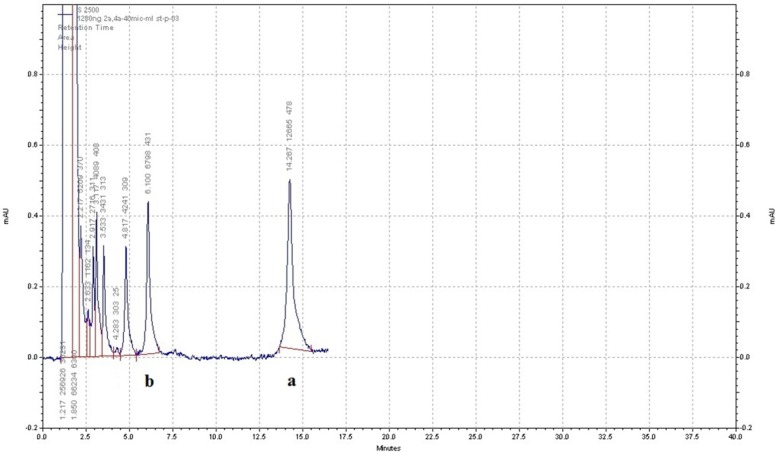
Sample HPLC chromatogram of AZGH101 (b) and diclofenac as internal standard (a) in plasma. The separation was done with buffer phosphate (10 mM) at pH = 2.7 and acetonitrile (50:50 (v/v)) as mobile phase and the flow rate of 1.5 – 2 mL/min in gradient mode

**Figure 4:**
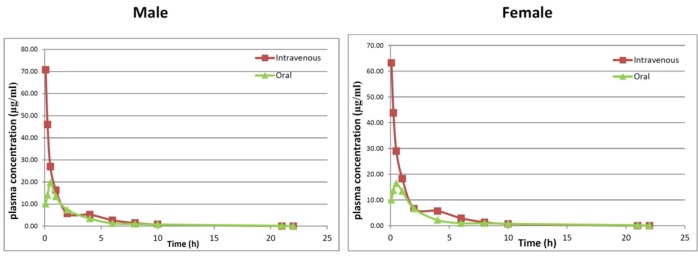
The plasma concentration versus time plots of AZGH101 after IV (10 mg/kg) and oral (20 mg/kg) administrations in male and female Wistar rats (6 rats/group

The stability study was conducted according to forced degradation protocol ICH guideline for new drug substance (14-16). In brief, the stability of AZGH101 at acidic, basic, and neutral medium was evaluated. Moreover, the effect of oxidative condition (H_2_O_2_, 3 and 30%), thermal degradation at solid state, and photolysis on the stability of AZGH101 was examined. 

*Pharmacokinetic Studies*

*Animals experiments*

Male and female Wistar rats weighting 250 ± 10 g were used. They were kept at 25 ± 1 ºC temperature and controlled humidity with 12 h light/dark cycle. The animals were fasted overnight before the experiment and had free access to water. The study protocol was approved by the local committee for animal experiments of Shahid Beheshti University of Medical Sciences (Tehran, Iran).

*Pharmacokinetic study*

Male or female rats were chosen randomly (6 rats/group) and assigned to receive AZGD 101 solution in normal saline for intravenous (IV) via the tail vein or oral administration via oral gavages. The administered doses in both sexes for intravenous (IV) and oral studies were 10 mg/kg and 20 mg/kg, respectively. Blood samples were collected from the tail vein immediately prior to administration (blank sample) and at 0.08, 0.25, 0.5, 1, 2, 4, 6, 8, 10, 21 and 22 h after administration into heparinized micro-tubes. Blood samples were centrifuged at 1000 g for 10 min and the separated plasmas were kept at –20 °C until analysis. 

*Pharmacokinetic analysis*

Pharmacokinetic analyses were performed using WinNonlin software (Pharsight Corporation, Mountain View, USA, Version 3.2) and two-compartmental model was chosen. The following pharmacokinetic parameters were estimated: terminal elimination half-life (t1/2), area under the plasma concentration versus time curve from zero to the infinity (AUC 0-∞), distribution and elimination rate constants (α, β), volume of distribution (Vd), and total body clearance (CL). The peak plasma concentration (C_max_) and the time to reach C_max_ (T_max_) for oral dose were obtained directly from the observed individual plasma concentration-time data. 

*Statistical* *analysis*

Data were shown as the mean ± standard deviation (SD). Statistical analyses were performed by using an unpaired *t*-test. A *p*-value of less than 0.05 was considered to be statistically significant. All calculations and statistical analysis were performed by Microsoft office excel software (2007). 

## Results and Discussion

*Structure and purity determination*

The obtained results for IR spectrum of synthesized AZGH101 were as follow: IR (KBr): ν (cm^-1^) 3514-2576 (OH), 1701, 1665 (C=O), 1314, 1152 (SO_2_). LCMS (ESI) chromatogram demonstrated a peak with 432.1 (M+1) ^+^ which was in agreement with the mass of AZGH101 (Mw: 431.08). The acquired data for elemental analysis was: Anal. Calcd for C_24_H_17_NO_5_S: C, 66.81; H, 3.97; N, 3.25. Found: C, 70.03; H, 3.79; N, 3.23 which were within ± 0.4% of theoretical values. These data demonstrated the high purity (99%) of purity for synthesized AZGH101. The λmax for AZGH101 was 270 nm in the UV spectrum (Figure. 2).

*Analytical method development*

The results indicated that changing the flow rate, mobile phase constituents, and mobile phase pH can affect the peaks shape, retention time, and area under the curve (AUC) of analyte. The AUC of analyte peak decreased when the flow rate increases. This effect was reported previously by Chen *et al*. (17). According to the peaks resolution and retention time of the analyte, appropriate flow rate was chosen. Substituting acetonitrile with methanol resulted in reduction of analyte retention time and column pressure as well as increasing the peak sharpness and resolution. Moreover, the pH of mobile phase especially at the range of 2.5-3.5 affected the retention time and peak sharpness. The results showed that the best condition for separation of AZGH101 from interfering peaks was the mixture of buffer phosphate (10 mM) at pH = 2.7 and acetonitrile (50:50 (v/v)) as mobile phase and the flow rate of 1.5 – 2 mL/min in gradient mode from 0 to 17 min. In this condition the retention time of AZGH101 was about 6 min. Among six proposed compounds (Celecoxib, piroxicam, meloxicam, naproxen, ibuprofen and diclofenac) as an IS; only diclofenac (50 ng/mL) had a suitable retention time of 15 min in the selected chromatographic condition (Figure. 3).

*Analytical method validation*

There were no interfering peaks in the chromatogram of blank (methanol), spiked samples with plasma, and blank plasma. The obtained results for precision, linearity, accuracy, LOD, and LOQ of AZGH101 in methanol and plasma are presented in Table 1. The obtained RSD in all concentrations were less than 3% which indicated that precision of this method was in a suitable range. The accuracy had a similar result, also.

The obtained LOD and LOQ for AZGH101 at methanolic medium was 4 and 12 ng/mL, respectively. Whereas the obtained LOD and LOQ for AZGH101 at plasma medium was about 7 times greater than methanolic medium and about 30 and 70 ng/mL, respectively. 

All calibration curves showed good linearity (r^2^>998) in the selected range either in methanol (20-320 ng/mL) or plasma (160-2560 ng/mL). The obtained slop for AZGH101 in methanol and plasma showed significant (*P* < 0.05) differences with the zero were. However, the intercepts showed no significant differences with zero (*P* > 0.05).

Recovery study demonstrated that in the optimum condition AZGH101 can be recovered up to 70.1 ± 1.1 %. The recovery percent for diclofenac as IS in similar condition was 72.5 ± 2.1.

*Physicochemical properties*

The reported melting point range for AZGH101 by Zarghi *et al*. was 246-248 °C (9). In this study the melting point of AZGH101 was 246 °C which was in the reported range. The obtained results demonstrated the aqueous solubility of AZGH101 was 1.4 ± 0.17 (mg/L) for 3 replications. It was reported that the solubility of ketoprofen and celecoxib (as selective COX-2 inhibitor) in water was 51 and 3.3 (mg/L), respectively. The aqueous solubility of this new derivative of ketoprofen was about one half of celecoxib. Another parameter, log P or logarithm of octanol/water partition coefficient, which is demonstrated the lipofilicity of compounds, was 3.82 ± 0.001 (n = 3) for AZGH101. The log P for celecoxib and ketoprofen are 3.4 and 3.1 respectively (18, 19). These data demonstrated that the lipophilicity of AZGH101 was similar or a few greater than the celecoxib and ketoprofen. Aqueous solubility and log P parameters demonstrated that AZGH101 can be considered as a lipophil compound. According to Lipinski›s rule of five, the drug molecule candidate should have log P less than 5. Moreover, the number of groups which donate and/or accept hydrogen atoms should be less than 5 and 10, respectively. Also, the molecular weight of drug molecule 

candidate should be less than 500 (20). So, the AZGH101 has suitable physico-chemical properties as a novel drug molecule. The results of stability study were shown in Table 2. As the data showed, in all of the examined conditions more than 90% of AZGH101 was recovered intact. This percent of recovery demonstrates the suitable stability of AZGH101.

*Pharmacokinetic study*

The plasma concentration versus time plots of AZGH101 after IV (10 mg/kg) and oral (20 mg/kg) administrations in male and female rats were shown in Figure 4. For both IV and oral administration plasma concentration-time data were best fitted with two-compartment model.

The calculated pharmacokinetic parameters of AZGH101 following IV and oral administration for both sexes were shown in Table 3.

*Pharmacokinetics of AZGH101 in rat following intravenous administration*

As shown in Table 3 following IV administration to male group, the elimination rate constant (β_male_) and distribution rate constant (α_male_) were 0.35 ± 0.01 (h^-1^) and 4.25 ± 0.99 (h^-1^), respectively. In female group, the β_female_ and α_female_ were 0.35 ± 0.01 (h^-1^) and 3.70 ± 0.80 (h^-1^), respectively. There was no significant difference between male and female groups (*P* > 0.05) for β and α parameters.

Apparent volume of distribution (Vd) which affects the total body clearance of the drug showed no differences between both sexes at IV route (89.26 ± 7.68 mL for female and 94.08 ± 6.42 mL for male). The values of Cl for both sexes were almost similar (Cl_male_ = 32.48 ± 1.57 mL/h and Cl_female_ = 31.63 ± 2.25 mL/h). Due to high lipophilicity of AZGH101, it is proposed that this compound excretes via bile duct. The double peak phenomenon which was shown in Figure. 4 may approve this hypothesis but more study is needed.

The AUC 0-∞ which is influenced by Vd and Cl showed no differences in both sexes (*P* > 0.05). Due to insignificancy in Cl and Vd between sexes, this result was expected for AUC. 

*Pharmacokinetics of AZGH101 in rat following Oral administration*

Following oral administration of AZGH101, the elimination rate constant (β_male_) and distribution rate constant (α_male_) were 0.14 ± 0.01 and 3.34 ± 1.26 (h^-1^), respectively. These parameters for female were as follow: β_female_ = 0.13 ± 0.01 (h^-1^) and α_female_ = 3.86 ± 2.99 (h^-1^). There was no significant difference between male and female groups (*P* > 0.05) for β and α parameters. However, α parameter was similar in both IV and oral route; β parameter at oral route was about one half of IV route. 

The observed Cmax in female and male groups was the same as well (C_max, male_ = 20.39 ± 1.49 μg/mL and C_max, female_ = 21.83 ± 1.33 μg/mL). As shown in Table 3, Vd and Cl values of AZGH101 showed no significant differences between both sexes at oral route (Vd: 245.87 ± 44.86 mL for female and 233.55 ± 27.82 mL for male, Cl_male_ = 32.45 ± 1.65 mL/h and Cl_female_ = 31.73 ± 3.31 mL/h). 

Based on the results, the oral absolute bioavailability of AZGH101 was about 27%, which was the same in both sexes (male 26.02 ± 0.03% and female 27.73 ± 0.01%; *P* > 0.05). According to the obtained data, the pharmacokinetic of AZGH101 was alike in female and male Wistar rats. Since the AZGH101 has higher lipophilicity than celecoxib and ketoprofen, the low bioavailability of this compound is predictable.

## Conclusion

The selective COX-2 inhibitor due to analgesic effect, anti-inflammatory effect and advantages in cancers and neurological diseases (2) can be used more than traditional NSAIDs. The AZGH101 was a new derivative of ketoprofen with selectivity index (COX-1 IC50 / COX-2 IC50) less than 500 which was suitable to introduce this compound as selective COX-2 inhibitors. AZGH101 according to Lipinski›s rule of five was suitable drug molecule candidate. The physicochemical properties of this new derivative indicated that this compound was lipophil. The pharmacokinetic parameters of this compound in Wistar rats demonstrated no significant differences between male and female sexes and were more similar to reported parameters for celecoxib than ketoprofen (18, 19). The cytotoxicity and pharmacokinetic study in other animal model maybe the next step in introducing this compound as new COX-2 inhibitors to the market. 

## References

[1] Moore RA, Chi CC, Wiffen PJ, Derry S, Rice AS (2015). Oral nonsteroidal anti-inflammatory drugs for neuropathic pain. Cochrane Database Syst. Rev.

[2] Zarghi A, Ghodsi R, Azizi E, Daraie B, Hedayati M, Dadrass OG (2009). Synthesis and biological evaluation of new 4-carboxyl quinoline derivatives as cyclooxygenase-2 inhibitors. Bioorg. Med. Chem.

[3] Dwivedi AK, Gurjar V, Kumar S, Singh N (2015). Molecular basis for nonspecificity of nonsteroidal anti-inflammatory drugs (NSAIDs). Drug Discov. Today.

[4] Green GA (2001). Understanding NSAIDs: from aspirin to COX-2. Clin. Cornerstone.

[5] Patrignani P, Patrono C (2015). Cyclooxygenase inhibitors: From pharmacology to clinical read-outs. Biochim. Biophys. Acta.

[6] Chandrasekharan NV, Dai H, Roos KL, Evanson NK, Tomsik J, Elton TS, Simmons DL (2002). COX-3, a cyclooxygenase-1 variant inhibited by acetaminophen and other analgesic/antipyretic drugs: cloning, structure, and expression. Proc. Natl. Acad. Sci. USA.

[7] Kim KJ, Choi MJ, Shin JS, Kim M, Choi HE, Kang SM, Jin JH, Lee KT, Lee JY (2014). Synthesis, biological evaluation, and docking analysis of a novel family of 1-methyl-1H-pyrrole-2,5-diones as highly potent and selective cyclooxygenase-2 (COX-2) inhibitors. Bioorg. Med. Chem. Lett.

[8] Zarghi A, Najafnia L, Daraee B, Dadrass OG, Hedayati M (2007). Synthesis of 2,3-diaryl-1,3-thiazolidine-4-one derivatives as selective cyclooxygenase (COX-2) inhibitors. Bioorg. Med. Chem Lett.

[9] Zarghi A, Ghodsi R (2010). Design, synthesis and biological evaluation of ketoprofen analogs as potent cyclooxygenase-2 inhibitors. Bioorg. Med. Chem.

[10] Cryer B, Feldman M (1998). Cyclooxygenase-1 and cyclooxygenase-2 selectivity of widely used nonsteroidal anti-inflammatory drugs. Am. J Med.

[11] OECD (1989). Guideline for Testing of Chemicals, no.117: Partition Coefficient (n-octanol/water), High Performance Liquid Chromatography Method.

[12] OECD (1995). Guideline for Testing of Chemicals, no.107: Partition Coefficient (n-octanol/water), Shake Flask Method.

[13] OECD (1995). Guideline for Testing of Chemicals, no.105: Water Solubility.

[14] International Conference on Harmonization (1999). ICH Specification: Test Procedures and Acceptance Criteria For New Drug Substances and New Drug Products (Q6A.

[15] International Conference on Harmonization (1999). ICH, Impurities: Impurities in New Drug Substances (Q3A).

[16] Bakshi M, Singh B, Singh A, Singh S (2001). The ICH guidance in practice: stress degradation studies on ornidazole and development of a validated stability-indicating assay. J. Pharm. Biomed. Anal.

[17] Chen CL, Uckun FM (2000). Highly sensitive liquid chromatography-electrospray mass spectrometry (LC-MS) method for the determination of etoposide levels in human serum and plasma. J. Chromatogr. B Biomed. Sci. Appl.

[18] National Center for Biotechnology Information (2015). PubChem Compound Database; CID=3825.

[19] National Center for Biotechnology Information (2015). PubChem Compound Database; CID=2662.

[20] Leeson P (2012). Drug discovery: Chemical beauty contest. Nature.

